# A Novel Microfluidic Cell Co-culture Platform for the Study of the Molecular Mechanisms of Parkinson's Disease and Other Synucleinopathies

**DOI:** 10.3389/fnins.2016.00511

**Published:** 2016-11-15

**Authors:** João T. S. Fernandes, Oldriska Chutna, Virginia Chu, João P. Conde, Tiago F. Outeiro

**Affiliations:** ^1^Instituto de Engenharia de Sistemas E Computadores (INESC) – Microsistemas e Nanotecnologias and Institute of Nanoscience and NanotechnologyLisbon, Portugal; ^2^Instituto de Medicina Molecular, Faculdade de Medicina da Universidade de LisboaLisbon, Portugal; ^3^Departamento de Bioengenharia, Instituto Superior Técnico, Universidade de LisboaLisbon, Portugal; ^4^Faculdade de Ciências Médicas, CEDOC – Chronic Diseases Research Center, Universidade Nova de LisboaLisbon, Portugal; ^5^Department of Neurodegeneration and Restorative Research, Center for Nanoscale Microscopy and Molecular Physiology of the Brain, University Medical Center GöttingenGöttingen, Germany

**Keywords:** microfluidics, Parkinson's disease, co-culture, cell culture, microglia, alpha-synuclein, inflammation

## Abstract

Although, the precise molecular mechanisms underlying Parkinson's disease (PD) are still elusive, it is now known that spreading of alpha-synuclein (aSyn) pathology and neuroinflammation are important players in disease progression. Here, we developed a novel microfluidic cell-culture platform for studying the communication between two different cell populations, a process of critical importance not only in PD but also in many biological processes. The integration of micro-valves in the device enabled us to control fluid routing, cellular microenvironments, and to simulate paracrine signaling. As proof of concept, two sets of experiments were designed to show how this platform can be used to investigate specific molecular mechanisms associated with PD. In one experiment, naïve H4 neuroglioma cells were co-cultured with cells expressing aSyn tagged with GFP (aSyn-GFP), to study the release and spreading of the protein. In our experimental set up, we induced the release of the contents of aSyn-GFP producing cells to the medium and monitored the protein's diffusion. In another experiment, H4 cells were co-cultured with N9 microglial cells to assess the interplay between two cell lines in response to environmental stimuli. Here, we observed an increase in the levels of reactive oxygen species in H4 cells cultured in the presence of activated N9 cells, confirming the cross talk between different cell populations. In summary, the platform developed in this study affords novel opportunities for the study of the molecular mechanisms involved in PD and other neurodegenerative diseases.

## Introduction

Parkinson's disease (PD) is a progressive neurodegenerative disorder characterized both by the loss of dopaminergic neurons in the substantia nigra pars compacta and the accumulation of intracellular protein inclusions primarily made up of alpha-synuclein (aSyn), known as Lewy bodies (Spillantini et al., 1997; Baba et al., 1998; Dauer and Przedborski, 2003; Goedert et al., 2012). Although, the precise molecular mechanisms underlying PD are still elusive (Obeso et al., 2010), sustained microglia activation and neuroinflammation seem to also contribute to the disease (Kim and de Vellis, 2005; Block et al., 2007; Long-Smith et al., 2009; Whitton, 2009; Glass et al., 2010). Several studies have suggested that aSyn aggregation and microglia activation might be connected, resulting in chronic neuroinflammation (Streit et al., 1999; Zhang et al., 2005; Su et al., 2008). Since it is known that aSyn is released and taken up by neighboring cells, thereby contributing to the progression of the disease (Lee et al., 2008), and that microglia sustains inflammation and damages surrounding cells mainly by releasing molecules such as cytokines and reactive oxygen species (ROS; Block et al., 2007), it would be important to investigate the interplay between these two processes using a platform that would enable the exchange of soluble molecules between different cell types. Such a platform would have to be able to replicate *in vivo* events where paracrine signaling is mostly dependent on diffusion and, at the same time, allow to quickly change the cellular microenvironment to provide cells with physical and/or chemical stimuli.

The use of cell models and traditional cell culture techniques enabled the isolation and replication of *in vivo* events to study both diseases and normal physiological processes. However, macroscopic cell culture techniques struggle to replicate events in which paracrine communication between different types of cells is key: tight spatial control over the cellular microenvironment and chemical stimuli are hard to achieve, the coexistence of diffusion, and convection make communication control and monitoring difficult, and when volumes are in the mL range the factors secreted by cells become diluted and ineffective. Microfluidic systems excel in the control and handling of both fluids and microenvironments (Toh et al., 2010; Young and Beebe, 2010; Mehling and Tay, 2014), due to the size scale, laminar fluid flow (Beebe et al., 2002), and the ability to pattern and modify the substrate where cells adhere (Kane et al., 1999; Rhee et al., 2005). Furthermore, microfluidic platforms equipped with integrated valves allow additional control, not only by permitting better fluid routing but also by offering the ability to keep defined sections of the platform isolated from other sections (Unger et al., 2000; Thorsen et al., 2002). This type of control makes microfluidics an excellent tool to study cell-cell communication by soluble factors, bridging the gap between *in vitro* and *in vivo*.

The advantages presented by microfluidic for cell culture are particularly useful for neurosciences and, as such, new platforms have been widely reported (Wang et al., 2009; Taylor and Jeon, 2010; Park et al., 2013). Some of these platforms present new solutions for very specific issues, from the study of axonal injury (Taylor et al., 2005) to the propagation of action potentials along axons (Dworak and Wheeler, 2009). The study of neurodegenerative diseases such as PD, in which paracrine signaling plays an important role, requires a platform that allows the co-culturing of different cell lines. Microfluidic devices designed for cell culture have been previously extensively reported (El-Ali et al., 2006; Mehling and Tay, 2014) but are usually either too complex for studying two cell populations or lack one or more desired features for this type of study. One platform has been reported that studies the interaction between microglia and neuroblastoma (Lovchik et al., 2009), but uses perfusion to ensure communication between the two populations of cells, not allowing to replicate diffusion-based events. Other recent platforms allow the study of diffusion-based events (Majumdar et al., 2011; Shi et al., 2013), but either do not ensure that cells are kept physically separated throughout the duration of the experiment—allowing for other types of communication such as juxtacrine signaling—or cannot manipulate the chemical environment of each cell population separately, the stimulation or specific activation of a given population for more complex studies. A device used to mimic the human gastrointestinal tract (Ramadan et al., 2013) allows the studying of the interaction between two co-cultured cell populations and the selective stimulation of each one, but since one population is cultured on top of a porous membrane true microenvironment separation cannot be achieved. Finally, a platform designed specifically for studying paracrine signaling by diffusion has been recently reported (Byrne et al., 2014), it relies on cell populations trapped in a 3D gel matrix, thereby complicating the dynamic manipulation of the microenvironment by perfusion. Although, all of these platforms successfully tackle their target application, to our knowledge no device is currently available that allows to study paracrine communication between physically separated cells all the while offering the possibility of dynamic and individual population stimulation and the keep isolated microenvironments for a user-defined amount of time.

In this study, we developed a novel microfluidic platform to study the communication between two different cell populations via soluble molecules. The platform is built around two cell culture chambers connected by three channels and equipped with integrated pneumatic valves that allow the cell populations to either be isolated from each other, or to be in communication via the exchange of soluble molecules and factors. The device is also designed in such a way that this communication can be established either by diffusion or by perfusion. In fact, since the distance between the two chambers is 250 μm, molecules released by one cell population can rapidly diffuse to the other, thereby mimicking *in vivo* conditions where cells are close together and paracrine signaling is efficient (Young and Beebe, 2010). Since the volumes used are in the nL range and the chambers are kept isolated from the rest of the platform, molecules remain confined in the cell culture area and do not diffuse to other areas of the device. Furthermore, since the device is made with transparent polydimethylsiloxane (PDMS), a versatile material that has been extensively used in biological and cell culture applications (Quake and Scherer, 2000; McDonald and Whitesides, 2002; Makamba et al., 2003; Sia and Whitesides, 2003; Mata et al., 2005; Berthier et al., 2012; Hegab et al., 2013; Xu et al., 2015), the platform is ideally suited for microscopy-based applications that afford the possibility of obtaining sub-cellular resolution in real-time and in living cells.

To demonstrate the usefulness of this platform and the relevance of monitoring cell-cell communication, we exploited molecular mechanisms associated with PD to conduct two proof-of-concept experiments: the study of the transmission of aSyn between two cell populations; and the impact of activated microglia cells on a neuron-like cell population. Although, co-cultures of neuron and microglia have been previously conducted (Lovchik et al., 2009; Majumdar et al., 2011; Shi et al., 2013), this new platform allows cells to communicate either by diffusion or by perfusion of molecules from one chamber to the other, while avoiding direct cell-cell contact throughout the duration of the experiments. In summary, this platform provides an important tool for replicating conditions closer to *in vivo*, while keeping a very tight control over the cells' microenvironment and their form of communication, and tracking their behavior in real time. The experimental venues made possible by this platform now enable future studies, namely focused on cell-cell interplay, that are extremely hard to achieve using current macroscopic technology.

## Materials and methods

### Device design and operation

**Figure 1:**
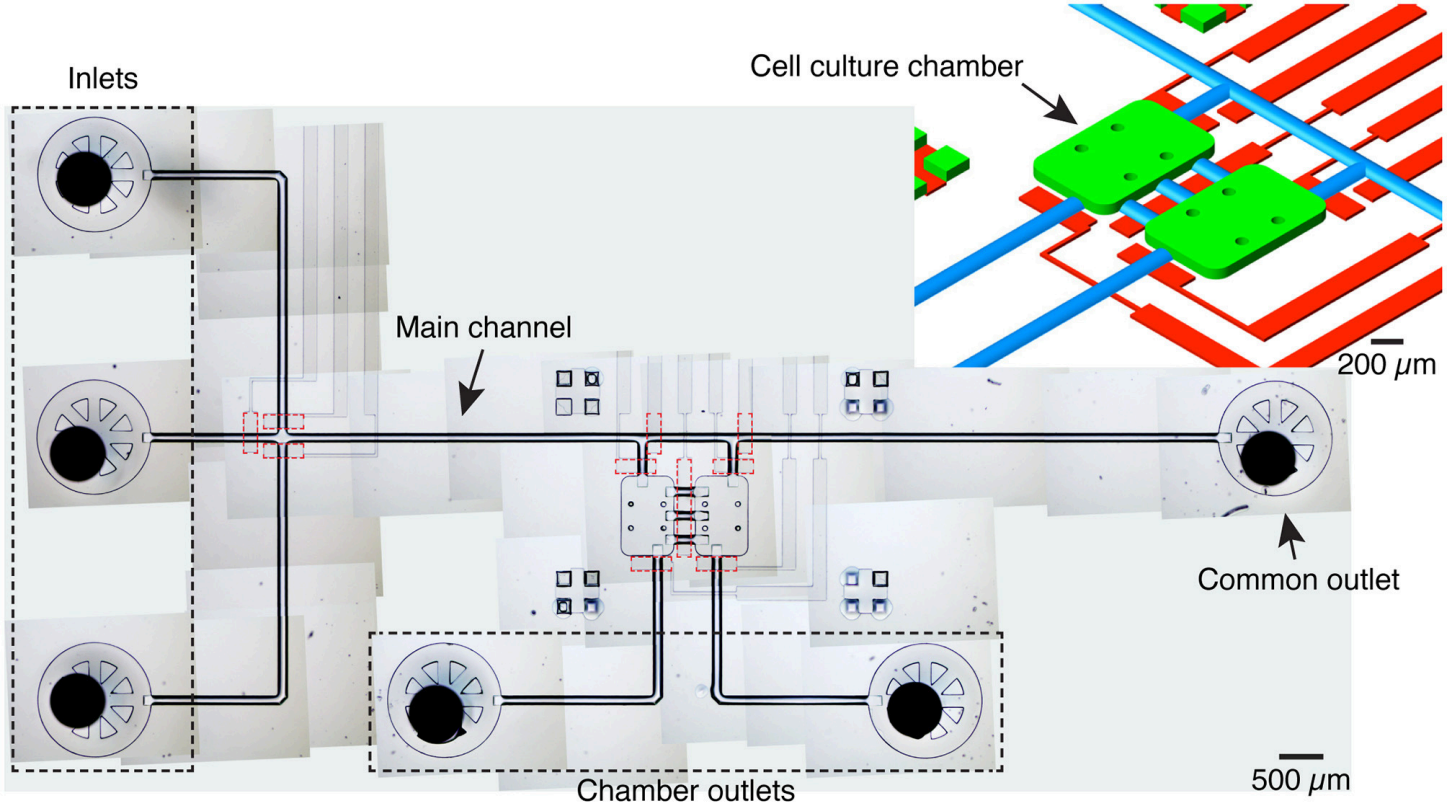
**Device overview**. Image of the microfluidic platform made by stitching several transmitted light microscopy images. The device includes 3 inlets, one main channel served by an outlet and two cell culture chambers with dedicated outlets. The cell culture chambers are connected to each other by three channels. Integrated pneumatic valves (dashed red lines) in a different PDMS layer allow to control fluid flow and to isolate inlets and cameras. A given cell culture chamber can be isolated by activating the valves at the entrance and exit, and the valve serving the central channels. Top right: schematic detail of the microfluidic structure with the cell culture chambers (green), pneumatic channels (red), and channels with a round cross-section (blue). For the mold of the flow layer, culture chambers, inlets and outlets were patterned with SU-8, while the channels with a round-cross section were patterned with AZ 40XT.

The microfluidic platform comprises two 600 × 900 × 70 μm chambers, each with a volume of 36 nL, linked by three 250 μm long, 125 μm wide channels (Figure 1). Three inlets are connected to a common channel that is connected to both chambers and ends in a common flush outlet. One inlet is dedicated to medium and the other two to different cell populations, thus avoiding cross-contamination. Each chamber also has a dedicated, independent outlet.

**Figure 2:**
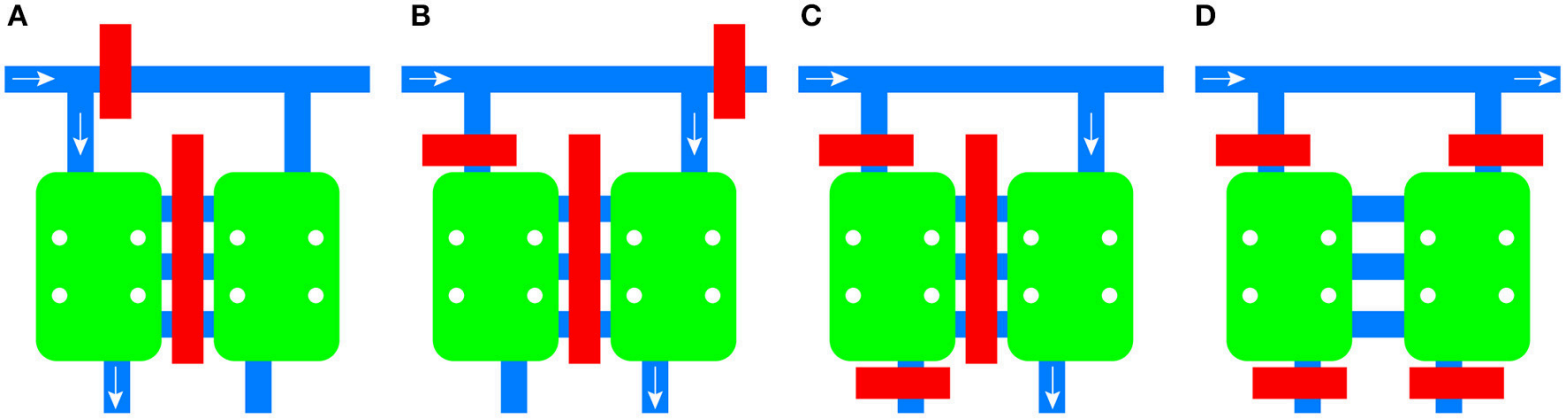
**Schematic of the flow control mechanism of the device**. Valves can be activated independently to route fluid to the left chamber **(A)** or the right chamber **(B)**. Individual chambers can be isolated from the rest of the platform **(C)** or both of them can be isolated while allowing diffusion between them **(D)**. Valves are shown in red, channels in blue, and cell culture chambers in green. The direction of the fluid flow is given by the white arrow.

Integrated pneumatic valves composed of 150 μm wide channels allow the routing of medium and cells to each chamber independently, the isolation of the microenvironment of each chamber, and the blocking of each inlet to the common channel (Figure 2). The channels between the chambers are also equipped with an integrated valve.

Before inserting cells, each device was sterilized and primed. Ethanol 70% was inserted into the microfluidic chip with a syringe pump (New Era Pump Systems, NY, USA) after which all inlets except one were plugged with a steel plug. Pressured air was then applied in the unplugged inlet at 80 kPa for 5 min to release all existing air bubbles (Kim et al., 2007). The channels were then rinsed with phosphate buffered saline (PBS) and coated with fibronectin (FN) to improve cell adhesion and proliferation (Klebe et al., 1981; Lee et al., 2004). The coating was performed by flowing a solution of 50 μg/mL of FN in PBS and incubating for at least 1 h at 37°C. Finally, before seeding the cells in the cell chambers, the device was rinsed with cell culture medium.

The pneumatic channels were also filled with DI water by filling the capillary tubing and applying about 200 kPa of pressure. By filling the channels with water one is able to effectively actuate the valves without running the risk of generating air bubbles in the medium by diffusion of gas through the PDMS membrane (Studer et al., 2004). The effectiveness of this type of valves was shown previously (Unger et al., 2000; Thorsen et al., 2002; Araci and Quake, 2012), and its leak-proof character was further verified with experiments using air bubbles and fluorescein isothiocyanate (data not shown).

Activation of the valves was controlled by switches connected to a simple custom-made printed circuit board (PCB; Supplementary Figure 1). The PCB was connected to a 24 V power source and the switches control small solenoid valves (SY144-5MZ-Q, SMC Corporation, Tokyo, Japan) mounted on a manifold, itself connected to a regulator and a pressured-air line. Each solenoid valve was linked to an integrated valve by polyethylene tubing (Instech Laboratories Inc., PA, USA).

### Fabrication

#### Mold

Two molds were required to build the microfluidic device: one for the fluidic layer, and a second for the pneumatic or control layer. Two different photoresists were used in the fabrication of the mold for the fluidic layer: SU-8 50 (MicroChem Corp., Newton, MA, USA) and AZ40 XT (MicroChemicals GmbH, Ulm, Germany), which are negative and positive photoresists respectively. This allowed to fabricate chambers and inlet punching marks with well-defined vertical walls using the negative photoresist and fluidic channels with a rounded cross-section, essential for the efficient operation of deflection-based pneumatic valves (Unger et al., 2000; Studer et al., 2004), by reflowing the positive photoresist. A 75 × 75 mm Si substrate was rinsed with de- ionized water (DI water), acetone and IPA and treated with 20 min of UV (UVO-Cleaner® 144AX, Jelight Company Inc., Irvine, CA, USA) to improve the substrate's wettability to SU-8 and prevent the regression and accumulation of the film toward the center of the substrate. SU-8 50 was spin coated at a final speed of 1500 rpm for 30 s, resulting in a final film thickness of about 70 μm. The film was processed according to the specifications of the manufacturer's datasheet, hard baked for 1 h at 180°C and left to cool down on the hot plate. This produced the cell chambers and punching marks for inlets and outlets. AZ40 XT was then dispensed on top of the Si substrate with the SU-8 features and left to settle for 4 min before spin coating at a final speed of 2000 rpm for 21 s and left again to settle for 30 min. These settling times are essential since the photoresist is viscous and tends to trap air bubbles between existing SU-8 features on the substrate, which are spread throughout the substrate during spin-coating. Soft-bake consisted of ramping the temperature from 100 to 125°C and baking for 5 min, followed by a 3 min cool down. A post-exposure bake at 105°C was performed for 2 min and the film was developed in AZ 400 K, a KOH-based developer. To obtain the required round cross-section, the AZ40 XT photoresist was reflowed in four steps: 5 min at 80°C, 5 min at 100°C, 15 min at 115°C, and 5 min at 125°C, with temperature ramping while the substrate was on the hotplate. After this treatment, channels with a rounded cross-section and a maximum height of 35 μm were obtained.

The pneumatic mold was fabricated with SU-8 2015: the substrate was first coated with a uniform layer of SU-8 2015 to facilitate PDMS release and the 20 μm features were patterned on a second layer according to the specifications of the manufacturer's datasheet.

#### Device

The device was fabricated with two PDMS layers (Sylgard 184, Dow Corning, Midland, MI) to allow the integration of pneumatic valves (Figure 1). The fluidic mold was placed into a poly(methyl methacrylate) (PMMA) case with a 5 mm thick spacer and a drilled lid. The several parts of the case were held together with metallic clips. Metallic connectors with a 23 ga diameter were inserted through the drills in the lid and aligned with the punching marks to form the access channels. PDMS at a 1:10 ratio was then inserted with a syringe through a hole placed on a corner of the lid until it filled the whole space and flowed out through another hole on the opposite corner. The bulk PDMS was then cured for 50 min at 70°C, after which the PDMS was released from the mold and the metallic connectors pushed through the bulk until they emerged on the other side ensuring that they went across the entire thickness of the PDMS. PDMS at a 1:20 ratio was spin-coated at 2200 rpm to obtain a thickness of about 30 μm on top of the pneumatic mold and cured for 40 min at 70°C. After curing, a small drop of ethanol was put on top of the 1:20 PDMS to serve as a lubricating layer to allow the alignment of both layers. It should be noted that, due to PDMS shrinkage (Lee and Lee, 2007; Moraes et al., 2009) the features of the mold for the bulk PDMS were scaled up by a factor of 1.012. After alignment the device was further cured for at least 1 h 30 min at 70°C and the access ports to the pneumatic layer were punched with a 20 ga luer stub. The device was then sealed against a glass coverslip (Hirschmann Laborgeräte GmbH & Co., Eberstadt, Germany) using oxygen-plasma bonding: both the device and the coverslip were exposed to oxygen-plasma (Harrick Plasma, Ithaca, NY, USA) for about 60 s, then brought into contact, creating a strong, irreversible bond (McDonald et al., 2000; Eddings et al., 2008). Finally, 22 ga right-angle stainless steel couplers (Instech Laboratories Inc., PA, USA) were inserted into the inlets and outlets and a drop of PDMS was applied to the interface of these ports with a syringe to create a liquid-proof sealing after curing for at least 30 min at 70°C.

### Cell culture conditions and transfections

Human H4 neuroglioma cells (HTB-148, LGC Standards, Barcelona, Spain) were cultured in Opti-MEM (Gibco, Thermo Fisher Scientific Inc, Waltham, MA, USA), supplemented with 10% heat inactivated fetal bovine serum (FBS, reference 10500-064, lot 07Q2330K, Gibco, Thermo Fisher Scientific Inc, Waltham, MA, USA) and 1% penicillin-streptomycin (pen-strep) and maintained at 37°C and 5% CO_2_. Before transfection, cells were plated in T-25 flasks and allowed to reach 70% confluence. Cells were transfected with wild type aSyn tagged with GFP (αSyn-WT-EGFP, WTSynEGFP plasmid previously described; McLean et al., 2001) or GFP (EGFP, pEGFP-N3 plasmid; Clontech, Palo Alto, CA, USA) using FuGENE (Promega Corporation, Wisconsin, USA). To select stably transfected populations, the medium was replaced with Opti-MEM complete medium supplemented with 400 μg/mL of G418 (Thermo Fisher Scientific Inc, Waltham, MA, USA) 24 h after transfection. A control flask containing non-transfected H4 cells was used to assess the efficacy of the selection. After the selection was completed, transfected cells were maintained in Opti-MEM complete medium supplemented with 200 μg/mL of G418.

N9 cells (a kind gift from Dr. Paula Ricciardi-Castagnoli) were cultured in RPMI 1640 (Gibco, Thermo Fisher Scientific Inc, Waltham, MA, USA) supplemented with 10% heat-inactivated FBS and 1% pen-strep. Activation of N9 cells was achieved by replacing the medium with RPMI 1640 complete medium supplemented with 1 μg/mL of lipopolysaccharides (LPS, from *Escherichia coli 055:B5*, chromatographically purified by gel filtration, Sigma-Aldrich, St. Louis, MO, USA) and incubating for at least 12 h.

### Image acquisition and processing

All images were acquired with a Nikon Eclipse Ti-E inverted microscope (Nikon Corporation, Tokyo, Japan) equipped with a motorized stage, a cage incubator for temperature control and a stage-top gas chamber for CO_2_ control (Okolab S.R.L., Pozzuoli, NA, Italy). Transmitted light and fluorescent images were acquired with a dry 10 × Nikon CFI Plan Fluor objective with a numerical aperture of 0.30 and a high sensitivity Andor iXon Ultra 897 EMCCD camera (Oxford Instruments, Oxfordshire, UK).

Acquired images were analyzed using ImageJ software (NIH, Bethesda, MD, USA) and stitched using the pairwise stitching plugin (Preibisch et al., 2009).

### H4 cell seeding and aSyn-GFP release

Cells in suspension were loaded in a syringe and inserted into the device at one of the inlets and a controlled flow was maintained by a syringe pump; liquid was allowed to flow through the common channel to the flush outlet until cells were seen entering the inlet; valves routed the cells into the chamber and the valve at the entrance of the chamber was opened and closed until the appropriate number of cells was inside the chamber. Since inertia does not play a significant role in this flow regime, the fluid flow stops immediately once the valve is closed and the number of cells can be quickly assessed. Cells were then allowed to sediment and adhere to the substrate for at least 90 min: it was seen that this was the necessary time for cells to spread on the substrate and acquire their typical epithelial-like morphology. It should be noted that several seeding rounds are possible: since cells are quick to sediment and adhere to the substrate in a few minutes, new cells in suspension can be routed into the chamber without detaching the previously seeded ones. Throughout the seeding process the channels linking both chambers were kept closed to prevent cross-contamination, and after the seeding of each cell population the common channel was rinsed with medium at a high flow rate (above 20 μL/min). Since during seeding the suspended cells are in constant perfusion through the common channel, and we can seamlessly switch the incoming flow from the cell to the medium inlets, cells do not have the chance to sediment and adhere to this channel, further minimizing the chance for cross-contamination.

**Figure 3:**
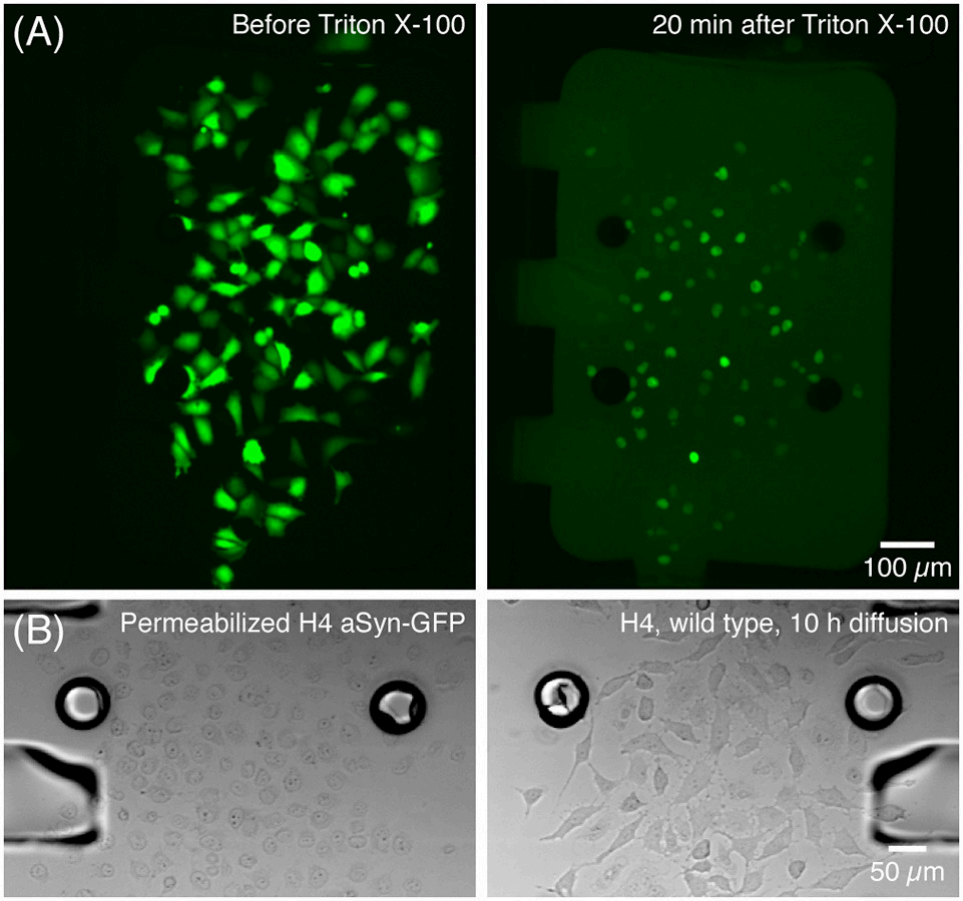
**Release of aSyn-GFP by treatment with Triton X-100. (A)** Cells producing aSyn-GFP (left) are permeabilized with 0.05% Triton X-100 in PBS and start releasing their contents to the medium in a matter of seconds (right). **(B)** Transmission microscopy images show that cells treated with Triton X-100 suffer significant morphological changes (left) and that 10 h after opening the central channel the wild type H4 cells are unaffected by Triton X-100 (right). This was also confirmed by viability staining with PI. Fluorescence images acquired with a 300 ms exposure.

After the static adhesion time, medium inside each chamber was renewed by flowing medium at 5 μL/min for 10–20 s—which was also used to flush non-adhered or dead cells. Two hours after insertion cells were already expressing aSyn-GFP, as evidenced by a strong fluorescence signal. At this time point, a solution of Triton X-100 (Panreac Química S.L.U., Barcelona, Spain) 0.05% in PBS was perfused for about 5 s at 5 μL/min in the cell chamber containing the cells with aSyn-GFP, after which the chamber was isolated by closing the valves at the entrance and exit. The cells were kept isolated for 20 min, after which the central channels were opened. This concentration of Triton was optimized so that it is sufficient to permeabilize the membranes of the cells inside the chamber, releasing aSyn-GFP in the medium, without affecting cells in the neighboring chamber once the valve is opened (Figure 3).

**Figure 4:**
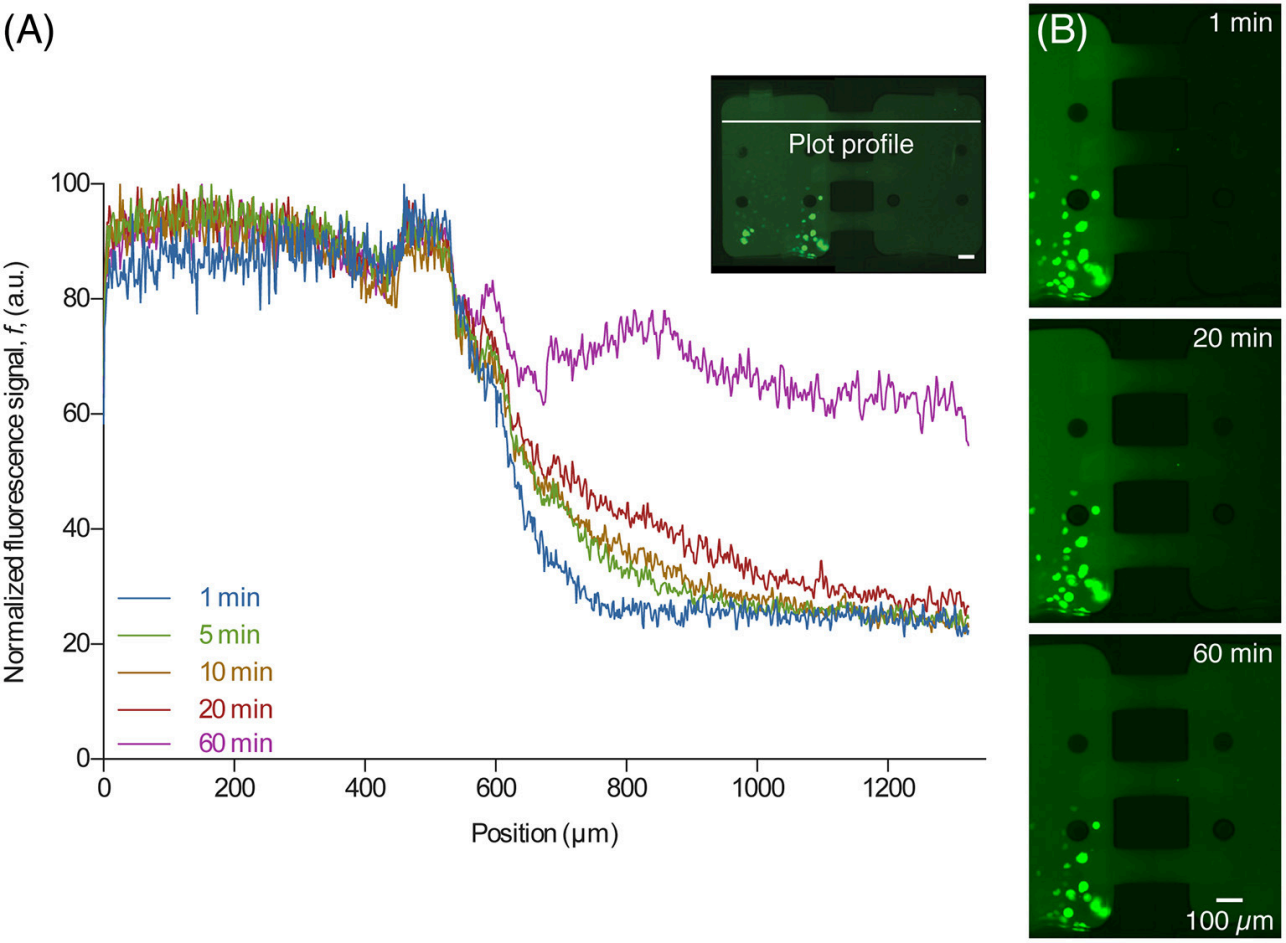
**Diffusion of released aSyn-GFP. (A)** An intensity plot profile of fluorescent signal across the two chambers for several time points shows that after 60 min the concentration of aSyn-GFP is almost the same in both chambers. The line used for data acquisition is shown on the image on the upper right corner (white line). **(B)** Fluorescence microscopy images taken 1, 20, and 60 min after opening the central channels, showing diffusion of aSyn-GFP from the left to the right chamber. Scale bars are 100 μm, fluorescence images acquired with a 300 ms exposure.

After the central channel was opened, aSyn-GFP diffused to the neighboring chamber (Figure 4). Both cell chambers were kept isolated but with the central channels open for 10 h, after which they were rinsed with PBS, fluorescent images were taken and a viability staining was performed. For the staining 10 μg/mL of propidium iodide (PI) was introduced in the device and left to incubate for about 20 min.

### N9 and H4 co-culture

For N9 and H4 co-culture, two sets of experiments were made: one with activated, and another with non-activated N9 cells. Seeding of both N9 and H4 was made as described above. In the assay with LPS-activated N9, previously activated cells were suspended in RPMI 1640 complete medium supplemented with 1 μg/mL LPS, while non-activated N9 were suspended in complete medium without LPS prior to seeding. Cells were left to settle and adhere for 2 h, after which medium was changed for both populations. In both sets of experiments, the fresh medium did not contain LPS. The central channel was opened 30 min after the medium change, and diffusion was allowed to occur for 10 h. At the end of the time allowed for diffusion, DHE at 30 μM in Opti-MEM complete medium was inserted in the chambers and left to incubate for 20 min at 37°C.

## Results and discussion

### aSyn spreading

The prion-like spreading of aSyn pathology in the brains of PD patients is intriguing, and the precise molecular mechanisms involved are still elusive (Li et al., 2008; Olanow and Prusiner, 2009). Here, we took advantage of the microfluidic platform we developed to study and monitor the transfer of this protein between two cell populations in real time. By co-culturing cell populations expressing or not expressing aSyn, we studied conditions that affected the release and uptake of the protein. For this purpose, we used a GFP-tagged version of aSyn (aSyn-GFP; previously described in McLean et al., 2001), to enable the imaging of the process in living cells and in real-time. In this particular proof-of-concept study we focused on the uptake of aSyn and, therefore, promoted the release of aSyn-GFP by permeabilizing the cell membrane.

To study the spreading of aSyn between cells (Lee et al., 2008; Lashuel et al., 2012) naïve H4 cells were cultured with H4 cells expressing aSyn-GFP in the two chambers of the device as described above. This cell line chosen since H4 cells and other types of neuroblastoma have been extensively used in the study of the release of aSyn and other proteins (El-Agnaf et al., 2003; Alvarez-Erviti et al., 2011; Herrera et al., 2011; Chutna et al., 2014).

To assess if the uptake of aSyn-GFP occurred, the fluorescence signal of naïve H4 cells was measured before the opening of the central valve and after a 10 h exposure to aSyn-GFP in solution followed by washing with PBS. Regions of interest (ROIs) corresponding to the cells' perimeter were defined using a transmitted light microscopy image and these were used to measure the signal intensity in the fluorescence microscopy image. To ensure that the ROIs captured the fluorescence signal of the entire cells, transmitted light and fluorescence images were captured at the same time point. Cells that stained positive for PI were discarded from this selection. Since other studies reported spreading of aSyn between cells in as little as 5 min (Lee et al., 2008), we surmised that 10 h was an appropriate time frame to observe uptake events.

Since cells change shape and divide throughout the duration of the experiment, it was not practical to track each single cell and evaluate the change in fluorescence signal. The method used to determine if aSyn-GFP entered the cells, either through diffusion or endocytosis, was to compare the average signal inside the cell to the background fluorescent signal inside the cell culture chamber.

**Figure 5:**
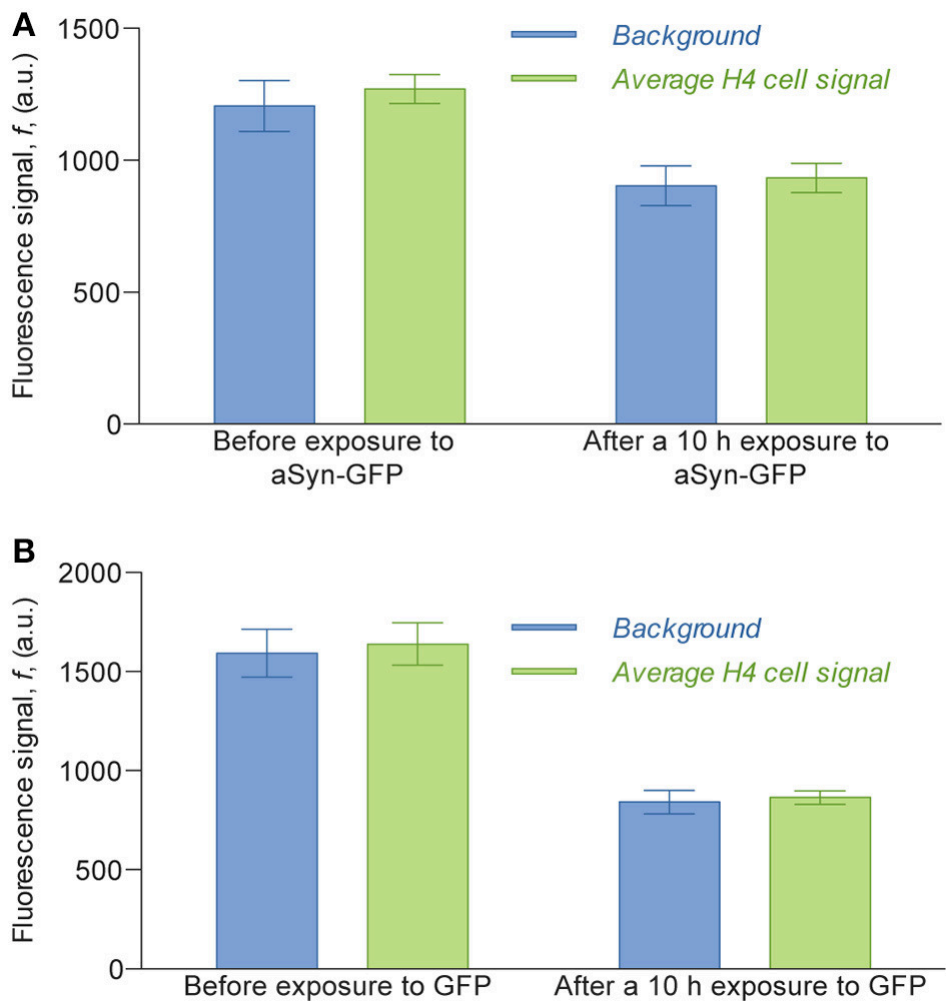
**Average fluorescence intensity signal of the H4 cells compared to the background fluorescence measured inside the cell chamber**. Fluorescence values of cells were obtained by selecting the areas corresponding to cells in images obtained with an epifluorescence microscope, taken with a 2 s exposure. For the assay with H4 aSyn-GFP **(A)**, the average signal before exposure was calculated counting 124 cells and the average signal 10 h after exposure was calculated over 116 cells; for the assay with H4 GFP **(B)** the numbers were 42 and 44, respectively. The ratio of cell signal to background fluorescence is 1.053 before exposure to aSyn-GFP and 1.033 after exposure **(A)** and 1.029 before exposure to GFP and 1.027 after exposure **(B)**. The difference between the background before and after exposure to aSyn-GFP or GFP can be explained by the fact that the fluorescence images of the latter were obtained after washing the device with PBS, which replaced the cell culture medium previously inside the cell chambers.

**Figure 6:**
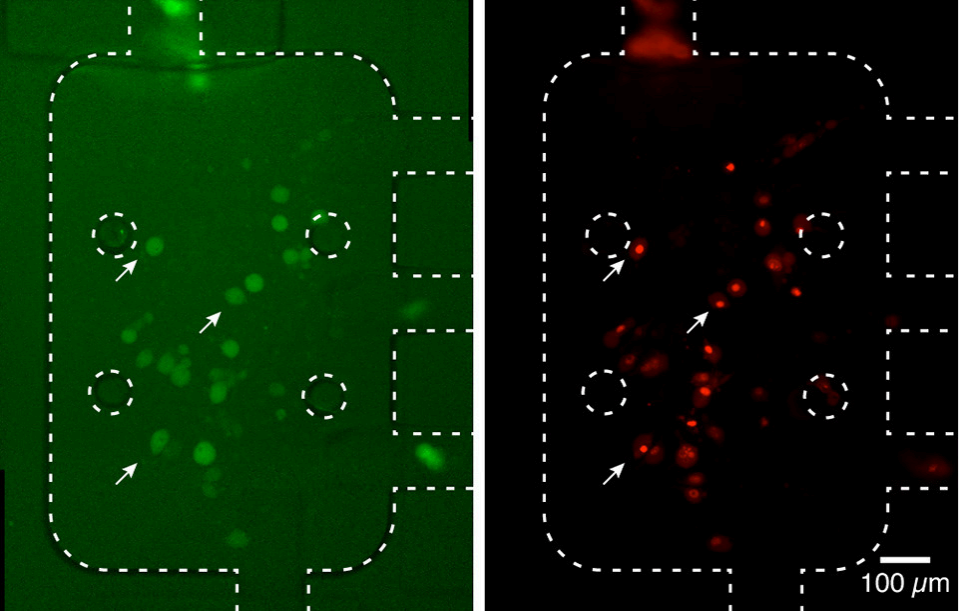
**Fluorescence signal in dead cells**. Cells with a higher fluorescence signal from aSyn-GFP, after washing with PBS (**left**, white arrows) displayed a compromised membrane after PI staining **(right)**. This seems to indicate that aSyn-GFP is able to penetrate and remain inside cells with compromised membranes. Similar results were obtained for H4 wild type cells co-cultured with H4 GFP cells. Fluorescence images were taken with 2 s **(left)** and 300 ms **(right)**.

Interestingly, in the experimental conditions used, we were not able to detect the uptake of aSyn-GFP (Figure 5A). In fact, although these cells have a slightly higher average fluorescence than the background (about 5%) this relation does change significantly after a 10 h exposure to aSyn-GFP. Although these observations seem to go against the general consensus of the literature, several possible explanations might justify these results: (i) the concentration of aSyn-GFP resulting from cell release is too low; (ii) aSyn molecules that find their way inside the cell might be rapidly degraded or re-released—in which case experiments would need to be carried out in a shorter time frame; (iii) molecules enter the cells in a very low number, and consequently have no visible influence on the fluorescence signal; (iv) GFP affects the uptake of or diffusion of aSyn, since it is a larger protein than aSyn itself. Control experiments in which H4 were co-cultured with H4 producing GFP seem to support the last explanation, since no uptake of GFP was observed (Figure 5B), however, other studies reported diffusion of GFP into cells in similar conditions (Reyes et al., 2015). It should be noted that in other studies using extracellular aSyn, internalization was observed after as little as 5 min for monomeric aSyn and 1 h for aSyn fibrils (Lee et al., 2008), which suggests that 10 h is an appropriate time scale for such an experiment. It is important to point out that a visible increase in fluorescence signal was found in cells that later showed positive PI staining, which would indicate that aSyn-GFP was able to enter cells with a compromised membrane (Figure 6). The same effect was verified with GFP alone. This could either indicate that active transport is necessary to constantly remove the molecule from inside the cell or that diffusion is less efficient across a non-compromised membrane. In any case, additional experiments will be required to clarify the mechanisms involved.

The use of aSyn tagged with fluorescent molecules has been used in various studies to monitor the uptake and release of this protein. However, these studies rely either on the use of previously prepared conditioned medium (Lee et al., 2008, 2010), i.e., medium in which cells released aSyn, or on culturing a populations expressing tagged aSyn together with another population expressing another fluorescent molecule (Reyes et al., 2015). This platform allows us to monitor the spreading of aSyn without the need for using recombinantly produced protein. Instead, we can directly monitor the release of cell-produced aSyn into the medium. The fact that we can perform all these steps inside the device increases the speed of the assay and improves reproducibility due to the simplification of the experimental variables. Furthermore, by using the platform we can also keep cells separated, thereby ensuring that any spreading happens via mechanisms that do not require cell-cell contact.

### Microglial cells affect the behavior of H4 cells

Since one of the main mechanisms through which microglia damages neurons is by the production of ROS, we used this microfluidic platform to monitor the effects of N9 microglia on naïve H4 cells. N9 microglial cells share many properties with primary microglia, namely activation by LPS and production of interleukin (IL)-6, tumor necrosis factor (TNF), IL-1, and ROS (Stansley et al., 2012) and are widely used in the research of neurodegenerative diseases. There are two main ways by which we can assess the impact of N9 on H4 cells: either by studying indirect effects such as changes in cell viability—which can be difficult to unequivocally relate to the presence of ROS or might not even occur if the concentration of ROS is small—or by directly evaluating the amount of ROS to which the cells are subjected using fluorescent dyes.

To demonstrate how microglia-neuron interactions can be studied in this platform, two sets of experiments were designed: on the first, H4 cells were co-cultured with non-activated N9 cells, as a control condition; on the second, H4 cells were co-cultured with LPS-activated N9 cells. The production of ROS and their effect on the H4 cells was evaluated by staining these cells with dihydroethidium (DHE), a fluorescent dye that becomes red and intercalates with DNA when oxidized by superoxide (Carter et al., 1994; Bindokas et al., 1996; Owusu-Ansah et al., 2008; Kalyanaraman et al., 2012).

Cell seeding was performed as described above and the fluorescence signal was evaluated by selecting ROIs corresponding to the stained nuclei of the cells in the fluorescence microscopy images.

**Figure 7:**
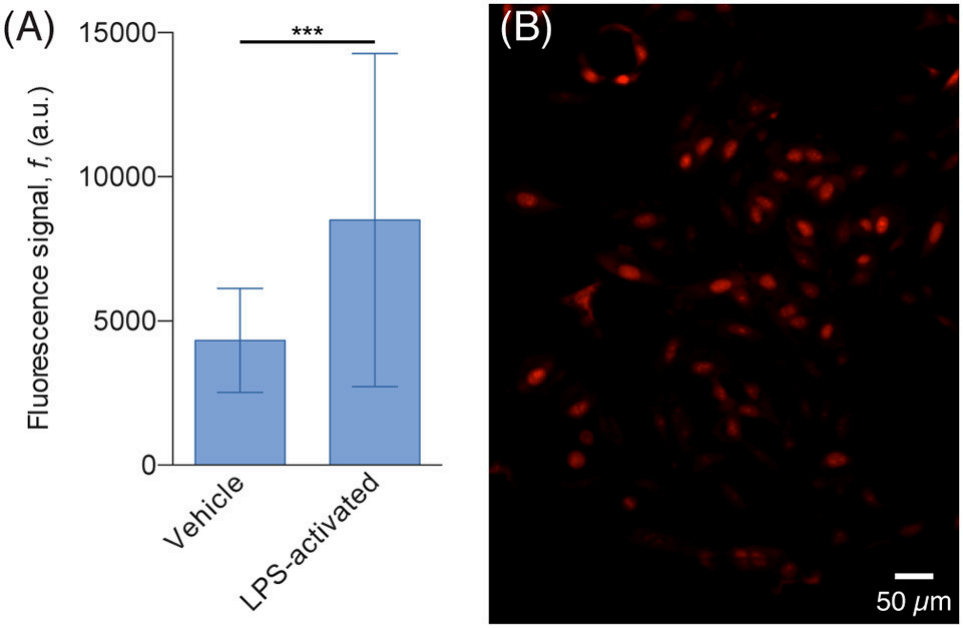
**H4 cells exhibit higher ROS levels when co-cultured with LPS-activated N9. (A)** Plot of the average DHE fluorescence signal of the H4 cells. The signal of cells co-cultured with LPS-activated N9 (32 cells, 77% of the population) is 97% higher than the one emitted from the cells co-cultured with non-activated (vehicle) N9 (74 cells, 78% of the population); ^***^p < 0.001 for two-tailed Student's *t*-test with unpaired data. **(B)** Fluorescence microscopy image of H4 cells, co-cultured with non-activated LPS, stained with DHE.

The results of the DHE assay showed that H4 cells co-cultured with activated N9 cells exhibit almost twice the fluorescence signal of cells co-cultured with non-activated N9 cells, which suggests that they are in the presence of higher levels of ROS (Figure 7). This difference cannot be explained merely by the difference in the number of N9 cells, since in the assay with non-activated cells the culture chamber contained 297 cells whereas in the assay with LPS-activated cells, there were 451 cells, which amounts to an increase of 52%. Furthermore, a two-tailed Student's *t*-test with unpaired data showed statistical significant difference from the mean signal of the two populations with *p* < 0.001. Although our observations produced the expected results, i.e., that LPS-activated N9 cells increased the overall level of ROS in H4 cells, more thorough analyses can be added to this type of preliminary study. Since cells are confined to microfluidic chambers, it is possible to know the exact number of both H4 and N9 cells—and not just an estimate as in traditional cell culture platforms. As such, it would be possible to find the relation between the number of N9 cells and the increase in DHE fluorescence signal. Furthermore, other ROS dyes could be used, such as 2′,7′-dichlorfluorescein-diacetate (DCFHDA; Macedo et al., 2014), and effects such as early apoptosis could be screened (Vermes et al., 1995; Datta et al., 2002) to see what other effects activated N9 have over the population. Although N9 cells pre-activated with LPS were used in this experiment, this platform allows the studying of the interplay of aSyn and microglia by stimulating resting microglia with aSyn, either released by cells or in supplemented medium. This will in turn allow replicate and monitor the mechanisms of sustained, chronic inflammation that occur *in vivo*.

## Discussion

In this study, a microfluidic platform was built with the aim of establishing a tool to study contactless cell-cell interaction, particularly in the context of synucleinopathies, such as PD. In particular, we focused on the assessment of the value of our platform for the study of aSyn spreading, a central problem in PD and also in other neurodegenerative disorders such as Alzheimer's disease (AD), where Tau pathology has also been show to spread in the brains of animal models (de Calignon et al., 2012) and between co-cultured cells *in vitro* (Frost et al., 2009). The platform we describe relies on two cell culture chambers where different cell populations can be seeded independently and the fluid flow can be easily and tightly controlled with a set of manual switches, thus altering their chemical microenvironment.

The applicability of this platform in the study of molecular mechanisms associated with synucleinopathies was demonstrated by two sets of proof-of-concept experiments addressing two major hallmarks of the disease: the spreading of aSyn and cell-cell communication between different cell types (neurons and glia). In particular, we focused on the interplay between neuronal and microglial cells, to study microglia-mediated neuroinflammatory responses.

In the first set of experiments, we showed that it was possible to obtain a very fine control over the cells' microenvironment by permeabilizing one cell population and leaving the other untouched. This approach released fluorescently-tagged molecules into the medium, which then diffused to the adjoining chamber. The set of integrated valves enabled the isolation of each chamber, thereby preventing the chemicals of interest from diffusing outside the chambers and being diluted. Cells survived 10 h without medium change and time-lapse imaging revealed cell motility and proliferation. Although we could not detect the uptake of aSyn-GFP, this device can be used to study transmission of proteins and other communication between cells exclusively through soluble molecules. This approach has the added advantage of releasing aSyn forms that naturally occur in cells, circumventing the need for adding purified recombinant proteins that are aggregated outside of the cellular environment and, therefore, less physiologically relevant. In the case of cells with high motility, extra steps can be taken to ensure that populations never come in direct contact with each other, namely by not coating the central channels with FN or even coating them with proteins that decrease cell adhesion such as bovine serum albumin (Klebe et al., 1981). Although, in this experimental protocol cells were lysed to release their contents, other approaches can be studied using this platform, such as tracking exocytosis of tagged aSyn aggregates using stressed cells or cells transfected with mutant aSyn.

In the second set of experiments, our goal was to observe, in real time, how cells with microglial phenotypes affected H4 cells. This was done by monitoring the diffusion of ROS produced by LPS-activated N9 cells in one chamber to the other chamber containing H4 cells via the positive staining of the H4 cells with DHE, an ROS dye. Given the control in fluid flow provided by this platform, it can also be used to expose N9 populations with different chemicals and observe their effect side-by-side—e.g., assess the kinetics of activation with different activators—and to replicate *in vitro*, with greater accuracy, events that occur during neuroinflammation. This second set of experiments aimed at exploring this device's capabilities to deliver venues of exploring ROS-based phenomena, and subsequent experiments in which exposure times and cell densities could provide interesting new data. Furthermore, additional techniques such as immunocytochemistry can also be used in this platform, which can be used to further study cell response to stimulus—e.g., the activation level of microglia when exposed to different concentrations of aSyn by using anti-Iba1 markers (Ito et al., 1998).

This platform also offers the possibility of studying chemotaxis. For example, it was observed that when cultured on their own, with no cells in the other chamber, non-activated N9 cells were highly motile but did not have a clear direction and did not cross to the other chamber (Supplementary Video 1). Future experiments can be designed to assess whether chemicals produced by a given cell population, such as aggregated aSyn (Wang et al., 2015), attract microglia: the existence of chemotaxis can be verified by comparing the number of cells that cross to the other chamber and comparing it to a control.

Although, co-culture platforms with pneumatic valves already exist, it is the number and position of the valves that makes this device highly versatile, since it allows not only bi-directional communication by diffusion, but also to establish one-sided communication by slowly perfusing cell culture medium from one chamber to the other. Although, the cell lines used in this work were useful for the proof of concept, and showed how *in vivo* events relevant to PD can be replicated on this platform, future studies of the molecular mechanisms of this disease should also involve primary cell cultures, but will involve future layers of optimization that go beyond the scope of the present study. In conclusion, our study established a novel microfluidic device enabling the study of cell-cell communication in tightly controlled conditions that will prove useful in many biomedically-relevant settings.

## Author contributions

All authors designed the research; JF and OC performed the experiments; JF, VC, JC, and TO wrote the paper.

## Funding

JF was supported by FCT (SFRH/BD/73908/2010). TO is supported by the DFG Center for Nanoscale Microscopy and Molecular Physiology of the Brain (CNMPB). The work was also supported by FCT through the Associated Laboratory IN—Institute of Nanoscience and Nanotechnology and the research project EXCL/CTM-NAN/0441/2012.

### Conflict of interest statement

The authors declare that the research was conducted in the absence of any commercial or financial relationships that could be construed as a potential conflict of interest.
