# Supplementary figures and images for: A Novel Microfluidic Cell Co-culture Platform for the Study of the Molecular Mechanisms of Parkinson's Disease and Other Synucleinopathies
Source: Front Neurosci. 2016 Nov 15;10:511. doi: 10.3389/fnins.2016.00511 (PMC5108800; doi:10.3389/fnins.2016.00511)

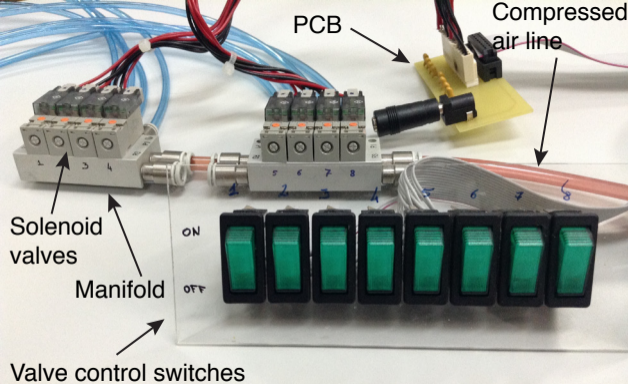

Supplement: Supplementary file 2 [file Image1.PDF]
